# Pulmonary function and symptoms in asthmatics adolescents

**DOI:** 10.1186/1939-4551-8-S1-A266

**Published:** 2015-04-08

**Authors:** Vitor Joaquim Barreto Fontes, Silvia De Magalhães Simões, Bárbara Bruna Fernandes De Andrade, Flávio Mateus Do Sacramento Conceição, Tamires Da Silva Fernandes, Ana Elisabeth Leal Varjão, Sérgio Luiz De Oliveira Santos, Jackeline Motta Franco, Mario Adriano Dos Santos

**Affiliations:** 1 Universidade Federal De Sergipe, Brazil; 2 Universidade Tiradentes, Brazil

## Background

Asthma is a chronic inflammatory disease that may impose limitations to daily activities when not appropriately treated. It can be under-diagnosed in adolescents due to possible denial of symptoms in this age group or low perception of airflow obstruction. The aim of this study was to measure the degree of airway dysfunction in adolescents with asthma and determine the degree of disease control.

## Methods

Asthmatic adolescents were identified in schools of Aracaju –Sergipe, based on the ISAAC questionnaire administered in 2012/2013. During the visits, spirometry was performed and the degree of disease control was evaluated through of specific questionnaire.

## Results

64 adolescents with asthma were identified, and 50 of them were interviewed. Spirometry was performed in 44 students. Most were from private schools, aged 12-14 years. Nineteen adolescents (38%) denied any manifestation of active asthma. Of the 44 patients who underwent spirometry, 29 (65%) reported symptoms of the disease and 19 (43%) had FEV1/FVC <0.80. Of these, 6 (31.6%) had no symptoms.

## Conclusions

Despite the trend towards higher frequencies of clinical manifestations in adolescents with airway obstruction, it is still possible to identify a percentage of asymptomatic adolescents in this group.

